# Draft Genome Sequence of *Klebsiella pneumoniae* AWD5

**DOI:** 10.1128/genomeA.01531-16

**Published:** 2017-02-02

**Authors:** Jina Rajkumari, L. Paikhomba Singha, Piyush Pandey

**Affiliations:** Department of Microbiology, Assam University, Silchar, Assam, India

## Abstract

Here, we report the draft genome sequence of *Klebsiella pneumoniae* strain AWD5, isolated from an automobile workshop in India. The *de novo* assembly resulted in a 4,807,409 bp genome containing 25 rRNA genes, 81 tRNAs, and 4,636 coding sequences (CDS). It carries important genes for polyaromatic hydrocarbon degradation and benzoate degradation.

## GENOME ANNOUNCEMENT

The genus *Klebsiella* belongs to the family *Enterobacteriaceae* and consists of seven species, including *Klebsiella pneumoniae*. *K. pneumoniae* is a free-living bacterium, which is found in soil, water, and a wide range of plants and animals, including humans. While *K. pneumoniae* is medically the most significant member of the *Klebsiella* genus of *Enterobacteriaceae*, it has been isolated from plants including rice and maize (1, 2) and from soil with the polyaromatic hydrocarbons bioremediation ability (3, 4).

Here, we report the draft genome sequence of *K. pneumoniae* strain AWD5, which was recovered from rhizospheric soil of a contaminated site in Silchar city, Assam, India. The strain was found to enhance the growth of plants and reduce the stress of the plants in the presence of polycyclic aromatic hydrocarbon (PAH) pollutants. Its genome was sequenced to study its genetic characteristics. The complete genomic DNA from *K. pneumoniae* AWD5 was sequenced using a Next Generation sequencing system. The sequencing libraries were prepared using a TruSeq DNA PCR-free sample preparation kit (Illumina, Inc., San Diego, CA, USA) following the manufacturer’s instructions. Using adaptive focused acoustic technology (AFA; Covaris), 1 mg of genomic DNA was fragmented and end-repaired to create 5′-phosphorylated, blunt-ended dsDNA molecules. This was followed by DNA size selection using a bead-based method. The purified libraries were quantified using qPCR according to the qPCR Quantification Protocol Guide (KAPA library quantification kits for Illumina Sequencing platforms). The libraries were qualified using the high-sensitivity DNA chip (Agilent Technologies, Waldbronn, Germany). Indexed libraries were then sequenced using the HiSeq2500 platform (Illumina, San Diego, USA). The bioinformatic analysis tools FastQC program and in-house perl script were used for the sequence quality check. Jellyfish was used for k-mer analysis in DNA. The sequences of the genome were assembled using SOAPdenovo2. The gene identification and annotation were provided by NCBI, conducted using the NCBI Prokaryotic Genome Annotation Pipeline, with the best-placed reference protein set and the GeneMarkS+ annotation method (https://www.ncbi.nlm.nih.gov/genome/annotation_prok/).

The total length of sequence was 4,807,409 bp. The assembly of *K. pneumoniae* AWD5 resulted in a genome, with a G+C content of approximately 65.96%. The genome was assembled into 137 contigs using CLC Genomics Workbench v9.0. The chromosome contains 25 copies of rRNA genes, 81 tRNA genes, and 4,636 protein-coding genes.

The strain has genes for dioxygenase to oxidize aromatic rings. A gene for catechol-1,2 dioygenase, which is a polyaromatic hydrocarbon-degrading enzyme was identified. Genes for benzoate-1,2 dioxygenase small and large subunits and a *benD* gene were also identified for benzoate degradation. The strain has the ability to release siderophores as confirmed by *in vitro* analysis and, accordingly, catechol types of siderophore genes were identified in the draft genome. The percentage of protein function found in the genome was 0.048% for energy production, 0.074% for amino acid transport and metabolism, 0.089% for carbohydrate transport and metabolism, 0.067% for general function prediction, and the cluster of orthologous groups (COG) of unknown function was found to be 0.061%.

### Accession number(s).

This whole-genome shotgun project has been deposited at GenBank under BioProject ID PRJNA326255 with accession no. MOXK00000000.

## References

[1] Andersen K, Shanmugam KT, Valentine RC 1977 Nitrogen fixation (NIF) regulatory mutants of *Klebsiella*: determination of the energy cost of N_2_ fixation *in vivo*. Basic Life Sci 9:95–110. 336030 10.1007/978-1-4684-0880-5_9

[2] Chelius MK, Triplett EW 2000 Immunolocalization of dinitrogenase reductase produced by *Klebsiella pneumoniae* in association with *Zea mays* L. Appl Environ Microbiol 66:783–787. doi:10.1128/AEM.66.2.783-787.2000. 10653751 PMC91896

[3] Afifi A, Motamedi H, Alizadeh B, Leilavi H 2015 Isolation and identification of oil degrading bacteria from oil sludge in Abadan oil refinery. Environ Exp Biol 13:13–18.

[4] Ping L, Zhang C, Zhang C, Zhu Y, He H, Wu M, Tang T, Li Z, Zhao H 2014 Isolation and characterization of pyrene and benzo[*a*]pyrene-degrading *Klebsiella pneumonia* PL1 and its potential use in bioremediation. Appl Microbiol Biotechnol 98:3819–3828. doi:10.1007/s00253-013-5469-6. 24389667

