# Erratum for Rajkumari et al., “Draft Genome Sequence of *Klebsiella pneumoniae* AWD5”

**DOI:** 10.1128/genomeA.01593-17

**Published:** 2018-01-25

**Authors:** Jina Rajkumari, L. Paikhomba Singha, Piyush Pandey

**Affiliations:** a Department of Microbiology, Assam University, Silchar, Assam, India

## ERRATUM

Volume 5, no. 5, e01531-16, 2017, https://doi.org/10.1128/genomeA.01531-16. Page 1, line 35: “65.96%” should read “58.18%.”

